# Motor Cortex-Periaqueductal Gray-Spinal Cord Neuronal Circuitry May Involve in Modulation of Nociception: A Virally Mediated Transsynaptic Tracing Study in Spinally Transected Transgenic Mouse Model

**DOI:** 10.1371/journal.pone.0089486

**Published:** 2014-02-19

**Authors:** Da-Wei Ye, Cheng Liu, Tao-Tao Liu, Xue-Bi Tian, Hong-Bing Xiang

**Affiliations:** 1 Cancer Center, Tongji Hospital of Tongji Medical College, Huazhong University of Science and Technology, Wuhan, Hubei, P. R. China; 2 Department of Anaesthesiology and Pain Medicine, Tongji Hospital of Tongji Medical College, Huazhong University of Science and Technology, Wuhan, Hubei, P. R. China; Inserm, France

## Abstract

Several studies have shown that motor cortex stimulation provided pain relief by motor cortex plasticity and activating descending inhibitory pain control systems. Recent evidence indicated that the melanocortin-4 receptor (MC4R) in the periaqueductal gray played an important role in neuropathic pain. This study was designed to assess whether MC4R signaling existed in motor cortex- periaqueductal gray- spinal cord neuronal circuitry modulated the activity of sympathetic pathway by a virally mediated transsynaptic tracing study. Pseudorabies virus (PRV)-614 was injected into the left gastrocnemius muscle in adult male MC4R-green fluorescent protein (GFP) transgenic mice (n = 15). After a survival time of 4–6 days, the mice (n = 5) were randomly assigned to humanely sacrifice, and spinal cords and brains were removed and sectioned, and processed for PRV-614 visualization. Neurons involved in the efferent control of the left gastrocnemius muscle were identified following visualization of PRV-614 retrograde tracing. The neurochemical phenotype of MC4R-GFP-positive neurons was identified using fluorescence immunocytochemical labeling. PRV-614/MC4R-GFP dual labeled neurons were detected in spinal IML, periaqueductal gray and motor cortex. Our findings support the hypothesis that MC4R signaling in motor cortex-periaqueductal gray-spinal cord neural pathway may participate in the modulation of the melanocortin-sympathetic signaling and contribute to the descending modulation of nociceptive transmission, suggesting that MC4R signaling in motor cortex- periaqueductal gray-spinal cord neural pathway may modulate the activity of sympathetic outflow sensitive to nociceptive signals.

## Introduction

Several studies have shown that motor cortex stimulation provided pain relief by motor cortex plasticity and activating descending inhibitory pain control systems[1], [2]_ENREF_2. Pagano et al [3] addressed that motor cortex stimulation produced its analgesic effects by enhancing neuronal firing rate and Fos immunoreactivity in the ipsilateral periaqueductal gray (PAG). Observations from Kerman et al suggested a novel role for the motor cortex and PAG in sympatho-motor integration[4]. A growing body of literature supports that sympathetic activity are tightly interconnected via central melanocortinergic pathways involving the melanocortin-4 receptor (MC4R)[5]–[7]. A previous report considered that the main cause of motor cortex stimulation- induced antinociception was due to opioid participation in PAG[8]. However, recent evidence indicated that melanocortinergic signaling in spinal cord and PAG played an important role in neuropathic pain[9]–[11].

A number of studies verified that the neurotropic pseudorabies virus (PRV) provided a highly specific method of mapping the motor and sympathetic pathways innervating a variety of targets [12]–[14]. The goal of the present study was to elucidate whether motor cortex-PAG-spinal cord pathway involved in the sympathetic neuronal circuitry by PRV-614 injection to the left gastrocnemius muscle in MC4R-GFP transgenic mouse. To prevent PRV-614 from being transmitted to motor cortex, PAG and spinal cord via motor circuitry, a spinal transection was performed just below the L2 level[15]. This study was designed to map the melanocortin-sympathetic pathway among motor cortex, periaqueductal gray and spinal cord in MC4R-GFP transgenic mice by using retrograde tracing techniques of PRV-614, and to assess whether MC4R signaling existed in motor cortex- periaqueductal gray- spinal cord neuronal circuitry that could modulate the activity of sympathetic pathway.

## Materials and Methods

### Animal Care

Transgenic MC4R-GFP mice, first obtained from Dr. Joel Elmquist and then bred to generate male and female mice, were genotyped as described by Rossi and colleagues[16]. All mice, weighing 25–30 g, were maintained in a standard 12-h light, 12-h dark cycle. Food and water were available *ad libitum*. All studies were performed following the National Guides for the Care and Use of Laboratory Animals and approved by the Institutional Animal Care and Use Committee of Tongji Hospital, Tongji Medical College, Huazhong University of Science and Technology University, Wuhan, China.

### Spinal transection and Postsurgical care

The L2 spinal cord was surgically transected in MC4R-GFP mice (n = 15) using a technique described previously [15]. Briefly, the skin overlying the dorsal process of the 13th thoracic vertebra was incised, the fascia and back muscles were dissected to expose the vertebra, and the dorsal aspect of the vertebra was removed using an electrical drill to expose the upper lumbar spinal cord. Subsequently, the spinal cord was transected just below the L2 level using an electrocautery. Transection was made carefully under magnification and the transection site subsequently cleaned with saline-soaked swabs. After surgical procedures were completed, back muscles, fascia, and the overlying skin was closed with surgical staples. The mice were provided with analgesia using a technique described previously [15]. Mice were provided analgesia with an intramuscular injection of a mixture of ketamine (10 mg/kg) and ketoprofen (3 mg/kg) just before the surgery (spinal cord transection) and every 12 h subsequently for a postsurgical period of 72 h. Following the surgery of spinal transection, animals had the paralysis of the lower body, and they were maintained in a special cage so as to have access to food and water. Gentle massage of the lower abdomen was operated for helping to urinate every 6 h after the spinal transection.

### Virus injection

The final titer was 2×10^8^ plaque-forming units/ml for PRV-614. Aliquots (20 µl) of the virus were kept in the freezer (−80°C), and on each experimental day, an aliquot was thawed and kept on ice until immediately before injections. Following our published procedures [17], [18], 3 d after spinal transection, a small incision in the skin overlying the left hindlimb muscle gastrocnemius was performed, and the muscle was bluntly dissected and separated from adjacent musculature and connective tissue. 2 µl injections of PRV-614 was injected into top parts of the gastrocnemius muscle [19] (0.5 µl per injection at 4 injection sites per mouse) using a 30-gauge needle connected to a Hamilton syringe (10 µl) under microscopic guidance. After each injection the muscle was swabbed with a cotton-tip applicator to decrease nonspecific viral spread. The time course of infection was empirically determined by carefully observing the pattern of infection at exactly 4 d (n = 5), 5 d (n = 5) and 6 d (n = 5) survival time.

### Tissue preparation and fluorescence immunohistochemistry

After a survival time of 4–6 days, mice (n = 15) were transcardially perfused as described previously[17], [18]. The brainstems and spinal cord were removed, post-fixed 2 hours, and submerged in 30% sucrose overnight at 4°C. The tissue was embedded in OCT compound and frozen on dry ice. Then, sections were cut at 30 µm using a cryostat and stored at 4°C until further processing. As a note, our study focused on the thoracic spinal cord, periaqueductal gray and motor cortex.

A band pass filter for Cy3 was used to identify cells infected by PRV-614. To determine which PRV-614-infected neurons coexpress MC4R-GFP, distinct fluorophores were used for GFP. The fluorescence immunohistochemical (IHC) procedure was carried out according to published protocols [20], [21]_ENREF_11_ENREF_1. For GFP IHC, first antibody used for immunohistochemical analysis was a chicken polyclonal antibody against GFP (ab13970, 1∶1,000; Abcam) and the corresponding second antibody included Alexafluor 488-conjugated anti-chicken IgG (A21200, 1∶1000; Abcam). After the completion of immunohistochemical processing, all sections were mounted onto slides, air dried, and cover slipped with mounting media.

### Tissue analysis

The sections were visualized by using an Olympus IX81 photomicroscope equipped with epifluorescence with a filter set for visualization. PRV-614- positive neurons are identified with red fluorescence, MC4R-GFP-expressing neurons are recognized by green fluorescence. Images were overlaid using Adobe Photoshop, and double-labeled neurons are presented as yellow (green/red). The regions in which positive cells were located were defined with reference to the atlases of Franklin KB and Paxinos G[22].

## Results

Injection of PRV-614 into the left gastrocnemius muscle resulted in uptake, self-amplifying, and transynaptic passage of the virus, and retrograde infection of neurons in the spinal cord and brain. In present study, we only examined neurons in the thoracic spinal cord, periaqueductal gray and motor cortex. We found that localization of the PRV-614 labeled neurons was unilateral and not bilateral in spinal cord. All mice infected with PRV-614 did not show apparent trait of illness or distress 4–6 d after virus injection.

### Specific expression of PRV-614 in the IML, PAG and motor cortex

**Figure 1:**
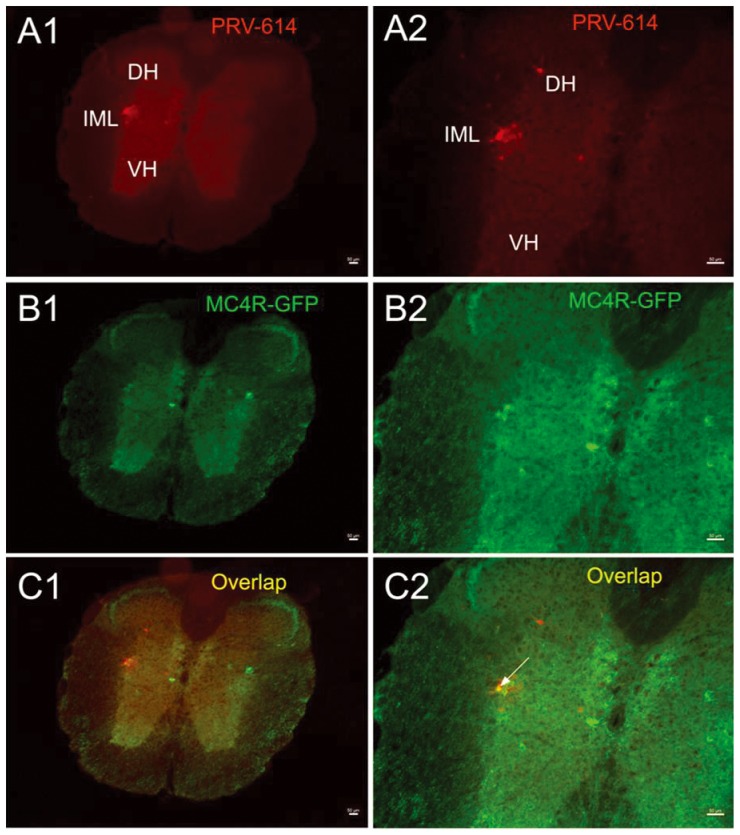
PRV-614/MC4R-GFP double-labeled neurons in the spinal cord. Images A1, B1, C1 were taken from an animal after injections of PRV-614. A**1** showed neurons infected with PRV-614, which send transsynaptic projections to the gastrocnemius muscle; B1 showed MC4R-GFP positive neurons in the spinal cord; C1 showed overlaid images of A1 plus B1. Images A2, B2, C2 amplified views of A1, B1, C1, respectively. Arrows indicate double-labeled neurons. In contrast to the IML, we didn't detect PRV-614 labeled neurons in the VH in spinally transected mice. IML, the intermediolateral cell column; DH, Dorsal horn; VH, ventral horn. Scale bars, 50 µm.

At 4 d survival time (n = 5), PRV-614 labeled neurons was located in the ipsilateral intermediolateral cell column (IML) of the lumbar and thoracic spinal cord (Fig. 1A). In contrast to the IML, we didn't detect PRV-614 labeled neurons in the ventral horn in all spinally transected mice (Fig. 1A), suggesting that animals received a successful spinal transection.

**Figure 2:**
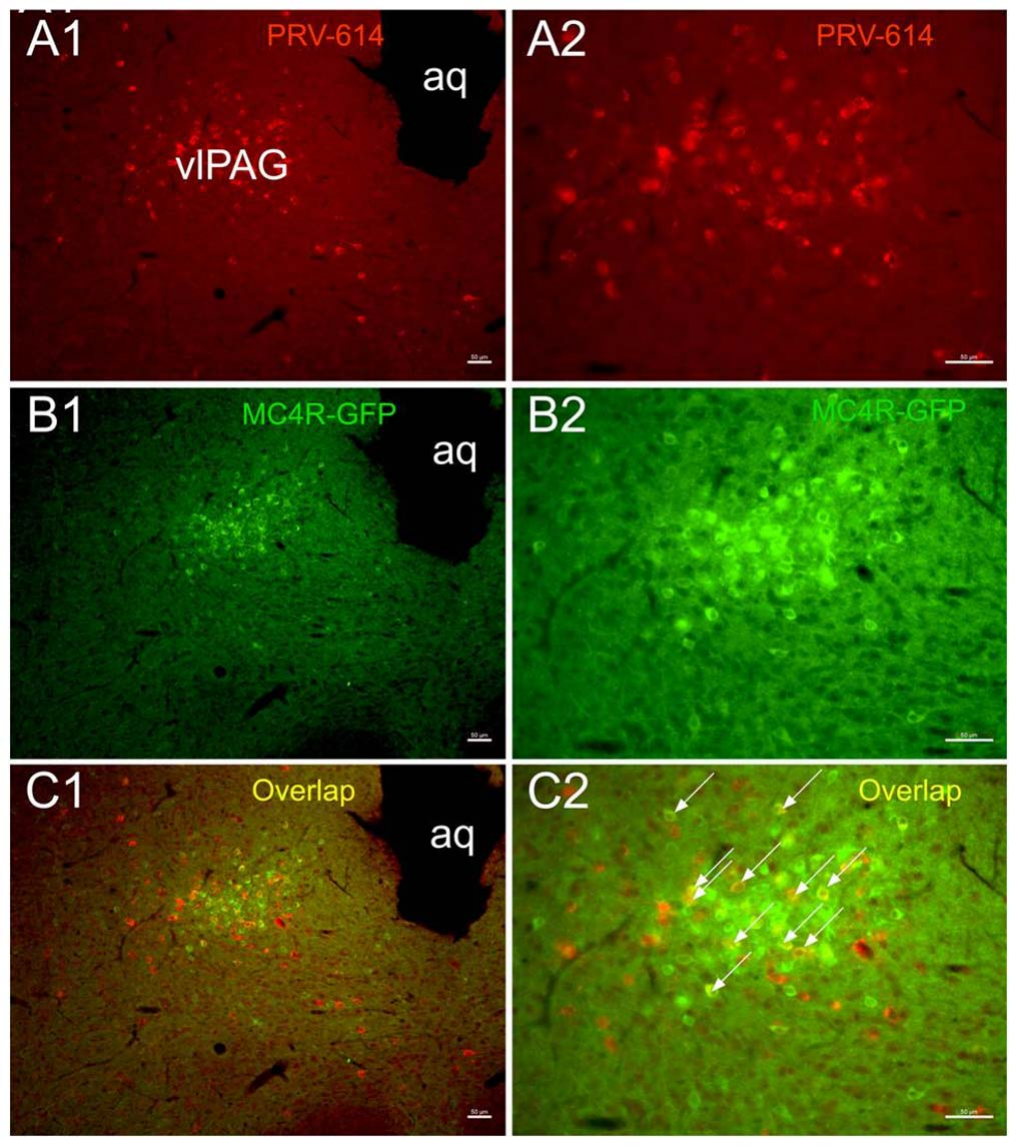
PRV-614/MC4R-GFP double-labeled neurons in the periaqueductal gray. Images A1, B1, C1 were taken from an animal after injections of PRV-614. A1 showed neurons infected with PRV-614, which send transsynaptic projections to the gastrocnemius muscle; B**1** showed MC4R-GFP positive neurons in the periaqueductal gray; C1 showed overlaid images of A1 plus B1. Images A2–C2 amplified views of A1–C1, respectively. aq, aqueduct; PAG, periaqueductal gray. Arrows indicate double-labeled neurons. Scale bars, 50 µm.

At 4∼5 d survival time, PRV-614 positive neurons were observed in the PAG. From 4 d to 5 d, the most substantial increase seen in the number of PRV-614-infected cells was in the PAG(5 d survival time, Fig. 2A). Additionally, two of the animals surviving 5 d began to show PRV-614 labeling in the motor cortex.

**Figure 3:**
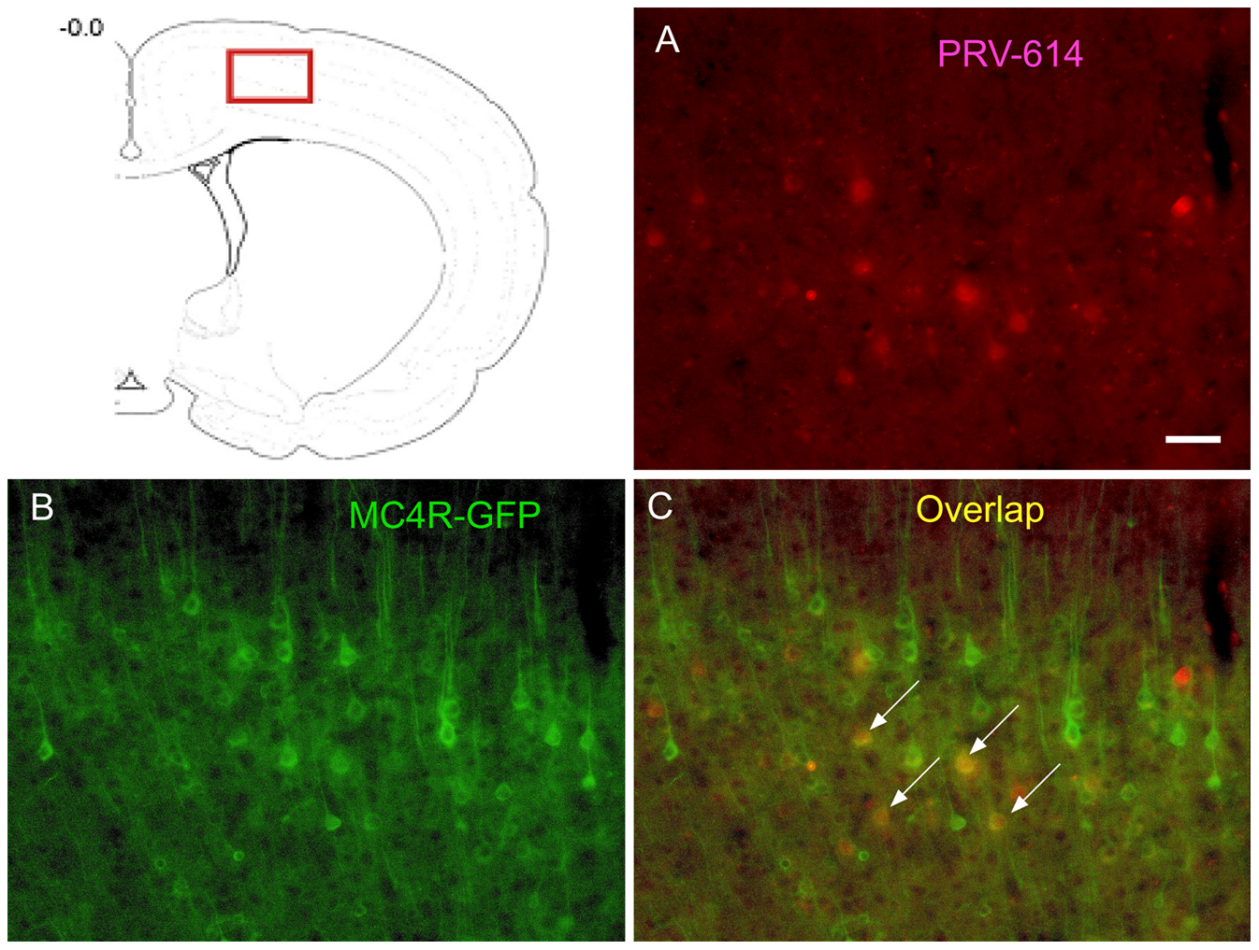
PRV-614/MC4R-GFP double-labeled neurons in the motor cortex. The red box in the top drawing indicates the approximate location from which the images were digitized. Images A–C were taken from an animal after injections of PRV-614. Image A showed neurons infected with PRV-614, which send transsynaptic projections to the gastrocnemius muscle; Image B showed MC4R-GFP positive neurons in the motor cortex; Image C showed overlaid images of A plus B. Arrows indicate double-labeled neurons. Scale bars, 50 µm. A drawing was taken from Ilan A. Kerman (J Neurosci 2006).

At 6 d survival time (n = 5), PRV-614 labeling in the motor cortex (Fig. 3A) was significantly increased in all animals.

### MC4R and PRV-614 co-expression in the IML, PAG and motor cortex

We assayed GFP expression in the MC4R-GFP reporter mouse and observed a large number of GFP-positive neurons in the IML, PAG and motor cortex. We found that PRV-614/MC4R-GFP dual labeled neurons were present in the IML of spinal cord (4 d survival time, Fig. 1C), PAG (5 d survival time, Fig. 2C) and motor cortex (6 d survival time, Fig. 3C).

## Discussion

We had characterized projections to the gastrocnemius muscle from the IML of spinal cord, PAG and motor cortex in MC4R-GFP transgenic mice by using retrograde tracing techniques of PRV-614, for direct visualization under fluorescence microscope. We found that injection of PRV-614 into the gastrocnemius muscle resulted in retrograde infection of neurons in the IML of spinal cord, PAG and motor cortex after spinal transection beneath L2 level. It is well known that the spinal L2 level is rostral to the motor neurons and sensory neurons innervating the gastrocnemius motoneurons[15], [19], and there is no evidence that the parasympathetic nervous system provides any innervations to limb muscles[15]. Because the spinal cord was transected rostral to the gastrocnemius motoneuron pool, the brain neurons were infected with PRV-614 via the sympathetic nervous system. Thereby, these data suggest that motor cortex- periaqueductal gray- spinal cord neural pathway may implicate in modulating the activity of sympathetic nervous system.

Report from Liu et al [23] had extended our knowledge of the distribution and function of the MC4R by the transgenic mouse line expressing green fluorescent protein under the control of the MC4R promoter, and contributed to our understanding of chemical phenotypes, electrophysiological responses and neural bases of MC4R-expressing cells. In agreement with Liu et al[23], we found that there were moderate numbers of MC4R-GFP positive neurons in spinal IML, PAG and motor cortex. It was a strikingly attractive that PRV-614/MC4R-GFP dual labeled neurons were also detected in the IML, PAG and motor cortex. It is well known that MC4R exerts a wide variety of functions with paramount importance in brain physiology. Recent studies have shown that MC4R played an important role in nociceptive behavior induced by nerve injury[11], and spinal MC4R may participate in regulation of central sensitization and morphine tolerance[24], [25]. Substantial evidence shows that PAG and rostral ventromedial medulla (RVM) are the key areas in descending nociceptive modulation and takes part in the transmission of spinal nociceptive information[9], [26], [27]. Based on all these findings, we speculate that MC4R signaling in motor cortex-periaqueductal gray-spinal cord neural pathway may contribute to the descending modulation of nociceptive transmission.

**Figure 4:**
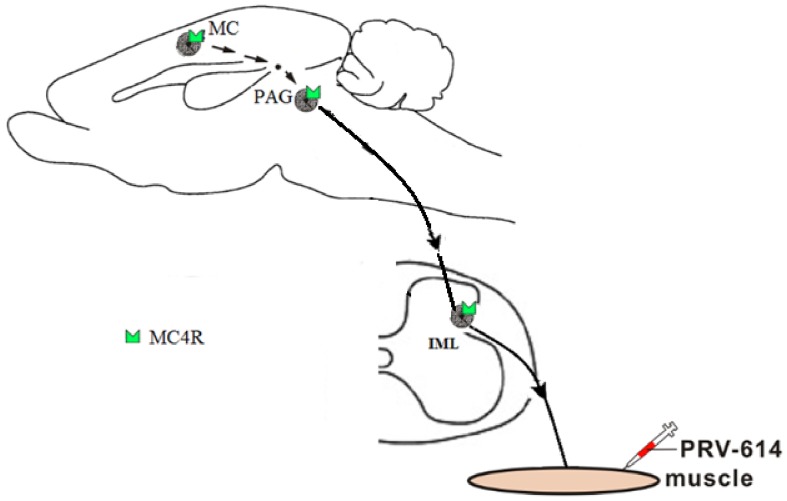
Summary diagram showed that the melanocortin-sympathetic pathway between the motor cortex and gastrocnemius muscle. Motor cortex, PAG and spinal cord are all participated in the modulation of the muscle activities. It is speculated that neurons within the motor cortex may send projections to PAG, which in turn projects to the IML sympathetic preganglionic neurons, which control gastrocnemius muscle activity by melanocortin-sympathetic signals. IML, intermediolateral column; MC, motor cortex; MC4R, the melanocortin-4 receptor; PAG, periaqueductal gray; PRV-614, pseudorabies virus-614. Some drawings were taken from HB Xiang (Brain 2013).

A number of studies had verified that motor cortex stimulation induced pain relief by activating descending inhibitory pain control systems[1], [2]_ENREF_2. Our reports supported this idea that the PRV-614/MC4R-GFP dual labeled neurons of motor cortex may influence sympathetic function in PAG and spinal cord. Our data further suggested that the motor cortex PRV-614/MC4R-GFP neuronal circuits involved in the regulation of sympathetic tone in PAG and spinal cord by melanocortinergic pathway, suggesting that stimulation of the motor cortex may influence sympathetic function in PAG and spinal cord by melanocortinergic pathway (Fig. 4). It should be noted that the mechanism of antinociception induced by motor cortex stimulation deserves further evaluation.

Taken together, these findings show that MC4R signaling in motor cortex-periaqueductal gray-spinal cord neural pathway may contribute to the descending modulation of nociceptive transmission, suggesting that that MC4R signaling in motor cortex- periaqueductal gray-spinal cord neural pathway may modulate the activity of sympathetic outflow sensitive to nociceptive signals. But further research is necessary that the involvement of the melanocortinergic and sympathetic systems needs to be evaluated in neuropathic rats submitted to the motor cortex stimulation.
